# Anthriscifolcine A, a C18-diterpenoid alkaloid

**DOI:** 10.1107/S1600536811001346

**Published:** 2011-01-15

**Authors:** Zhi-Jun Song, Wei-Quan Chen, Xiao-Ying Du, Yun-Fei Yuan, Han-Hong Xu

**Affiliations:** a Key Laboratory of Natural Pesticides and Chemical Biology, South China Agricultural University, Guangzhou 510642, People’s Republic of China; b College of Agriculture, Yangtze University, Jingzhou, Hubei 434025, People’s Republic of China

## Abstract

The title compound, C_26_H_39_NO_7_, which was isolated from *Delphinium anthriscifolium* var. majus, has a lycoctonine carbon skeleton containing four six-membered rings (*A*, *B*, *D* and *E*) and three five-membered rings (*C*, *F* and *G*). Rings *A*, *B* and *E* adopt chair conformation, while ring *D* adopts a boat conformation. Rings *C* and *F* adopt envelope conformations.

## Related literature

For the preparation, see: Song *et al.* (2007 ▶). For other lycoctonine-type diterpenoid alkaloids, see: Tashkhodjaev & Sultankhodjaev (2009 ▶). 
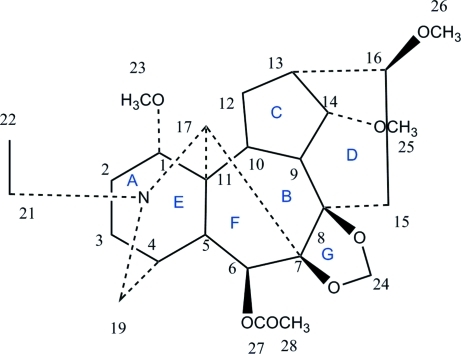


## Experimental

### 

#### Crystal data

C_26_H_39_NO_7_
*M* *_r_* = 477.58
Orthorhombic, 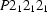

*a* = 8.9444 (16) Å
*b* = 14.135 (3) Å
*c* = 19.112 (3) Å
*V* = 2416.4 (7) Å^3^
*Z* = 4
Mo *K*α radiation
μ = 0.09 mm^−1^
*T* = 133 K
0.45 × 0.43 × 0.31 mm

#### Data collection

Rigaku AFC10/Saturn724+ diffractometer
19113 measured reflections
3122 independent reflections
3020 reflections with *I* > 2σ(*I*)
*R*_int_ = 0.034

#### Refinement

*R*[*F* ^2^ > 2σ(*F* ^2^)] = 0.034
*wR*(*F* ^2^) = 0.080
*S* = 1.00
3122 reflections
312 parameters
H-atom parameters constrained
Δρ_max_ = 0.24 e Å^−3^
Δρ_min_ = −0.17 e Å^−3^

### 

Data collection: *CrystalClear* (Rigaku, 2002 ▶); cell refinement: *CrystalClear*; data reduction: *CrystalClear*; program(s) used to solve structure: *SHELXS97* (Sheldrick, 2008 ▶); program(s) used to refine structure: *SHELXL97* (Sheldrick, 2008 ▶); molecular graphics: *SHELXTL* (Sheldrick, 2008 ▶); software used to prepare material for publication: *SHELXL97*.

## Supplementary Material

Crystal structure: contains datablocks I, global. DOI: 10.1107/S1600536811001346/bt5455sup1.cif

Structure factors: contains datablocks I. DOI: 10.1107/S1600536811001346/bt5455Isup2.hkl

Additional supplementary materials:  crystallographic information; 3D view; checkCIF report

**Fig. 1.**
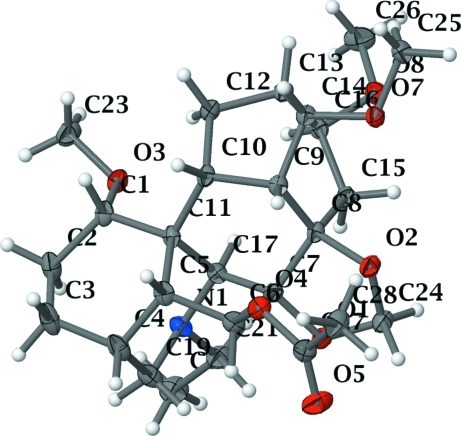
The molecular structure of the title compound with the atom numbering, showing displacement ellipsoids at the 30% probability level.

Enhanced figure: interactive version of Fig. 1

## supplementary crystallographic information

### Comment

The title compound, Anthriscifolcine A. (Song *et al.*, 2007), C_26_H_39_NO_7_, is a norditerpenoid alkaloid and has a lycoctonine carbon skeleton. It contains four six-membered rings (A, B, D and E) and three five-membered rings (C, F and G). Its structure is similar with other lycoctonine-type diterpenoid alkaloids beside the appearance of new five-membered ring, which formed by a methylenedioxyl group at C-7 and C-8. (Tashkhodjaev & Sultankhodjaev, 2009). The rings A, B and E adopt a chair-conformation, while ring D adopts a boat-conformation with C-8, C-9, C-13 and C-16 in the same plane. The five-membered rings C and F adopt envelope conformations with C-9, C-10, C-12 and C-13 of ring C in the same plane, as well as C-5, C-6, C-7 and C-17 of ring F in the same plane. Ring G adopts a significant distorted five-membered ring. Since this type of norditerpenoid alkaloid is a well known skeleton mainly originated from Delphinium and Aconitum Linn with the absolute configuration of C(5) being R, the absolute structure of the chiral carbon centres of the title compound are identified as 1*S*, 4*R*, 5*R*, 6*S*, 7*S*, 8*R*, 9*R*, 10*R*, 11*S*, 13*R*, 14*S*, 16*S* and 17*S*.

At first, through one-dimensional and two-dimensional NMR analysis, we supposed the title compound that was isolated from Delphinium anthriscifolium var. majus is an isomer of Anthriscifolcines A (Song *et al.*, 2007) for the strong 1*H*-^1^H COSY correlation of H-17 /H-5 seemingly indicative of the connection C(17)—C(5). On the other hand, comparison with the ^13^C NMR data reported by Dr Song, the chemical shift values of C-5, C-13, C-9 and C-10 differ from those of the one we got in D. A. var. majus. But X-ray crystal diffraction indicated that either the plane structure or its chirality was quite the same as Anthriscifolcine A as reported by Dr.  Song. After detailed analysis of the HSQC and HMBC spectra, we found that the chemical shift values of C-5 with C-13, as well as C-9 with C-10 were wrongly exchanged in the literature.

### Experimental

The title compound was isolated from the EtOAc fraction of the leaves of D. A. var. majus by a known method (Song *et al.*, 2007). Colourless single crystals were obtained by slow evaporation of an ethanol solution at room temperature (m.p. 485–487 K).

### Refinement

All H atoms were positioned geometrically and allowed to ride on their parent atoms at distances of C*sp*^2^—H = 0.93 Å with *U*_iso_=1.2*U*_eq_(C), and C*sp*^3^—H = 0.96 or 0.97 Å with *U*_iso_=1.5*U*_eq_(C).

### Crystal data

**Table:**

C_26_H_39_NO_7_	*D*_x_ = 1.313 Mg m^−^^3^
*M**_r_* = 477.58	Mo *K*α radiation, λ = 0.71073 Å
Orthorhombic, *P*2_1_2_1_2_1_	Cell parameters from 8028 reflections
*a* = 8.9444 (16) Å	θ = 3.1–27.5°
*b* = 14.135 (3) Å	µ = 0.09 mm^−^^1^
*c* = 19.112 (3) Å	*T* = 133 K
*V* = 2416.4 (7) Å^3^	Block, colorless
*Z* = 4	0.45 × 0.43 × 0.31 mm
*F*(000) = 1032	

### Data collection

**Table:**

Rigaku AFC10/Saturn724+ diffractometer	3020 reflections with *I* > 2σ(*I*)
Radiation source: Rotating Anode	*R*_int_ = 0.034
graphite	θ_max_ = 27.5°, θ_min_ = 3.1°
Detector resolution: 28.5714 pixels mm^-1^	*h* = −11→11
φ and ω scans	*k* = −18→18
19113 measured reflections	*l* = −21→24
3122 independent reflections	

### Refinement

**Table:**

Refinement on *F*^2^	Primary atom site location: structure-invariant direct methods
Least-squares matrix: full	Secondary atom site location: difference Fourier map
*R*[*F*^2^ > 2σ(*F*^2^)] = 0.034	Hydrogen site location: inferred from neighbouring sites
*wR*(*F*^2^) = 0.080	H-atom parameters constrained
*S* = 1.00	*w* = 1/[σ^2^(*F*_o_^2^) + (0.0431*P*)^2^ + 0.616*P*] where *P* = (*F*_o_^2^ + 2*F*_c_^2^)/3
3122 reflections	(Δ/σ)_max_ < 0.001
312 parameters	Δρ_max_ = 0.24 e Å^−^^3^
0 restraints	Δρ_min_ = −0.17 e Å^−^^3^

### Special details

**Table:**

Geometry. All e.s.d.'s (except the e.s.d. in the dihedral angle between two l.s. planes) are estimated using the full covariance matrix. The cell e.s.d.'s are taken into account individually in the estimation of e.s.d.'s in distances, angles and torsion angles; correlations between e.s.d.'s in cell parameters are only used when they are defined by crystal symmetry. An approximate (isotropic) treatment of cell e.s.d.'s is used for estimating e.s.d.'s involving l.s. planes.
Refinement. Refinement of *F*^2^ against ALL reflections. The weighted *R*-factor *wR* and goodness of fit *S* are based on *F*^2^, conventional *R*-factors *R* are based on *F*, with *F* set to zero for negative *F*^2^. The threshold expression of *F*^2^ > σ(*F*^2^) is used only for calculating *R*-factors(gt) *etc*. and is not relevant to the choice of reflections for refinement. *R*-factors based on *F*^2^ are statistically about twice as large as those based on *F*, and *R*- factors based on ALL data will be even larger.

### Fractional atomic coordinates and isotropic or equivalent isotropic displacement parameters (Å^2^)

**Table:**

	*x*	*y*	*z*	*U*_iso_*/*U*_eq_	
O1	0.30623 (14)	0.49917 (9)	0.71667 (6)	0.0201 (3)	
O2	0.44209 (14)	0.42792 (9)	0.62909 (7)	0.0203 (3)	
O3	0.72126 (15)	0.46178 (8)	0.91841 (6)	0.0199 (3)	
O4	0.36463 (14)	0.26667 (8)	0.70942 (7)	0.0195 (3)	
O5	0.13732 (16)	0.30454 (11)	0.66750 (8)	0.0329 (3)	
O7	0.77283 (14)	0.39651 (9)	0.59355 (6)	0.0194 (3)	
O8	0.85214 (15)	0.60530 (9)	0.65703 (7)	0.0232 (3)	
N1	0.37859 (17)	0.49307 (10)	0.87482 (8)	0.0191 (3)	
C1	0.6416 (2)	0.37444 (12)	0.90962 (9)	0.0197 (4)	
H1	0.7167	0.3220	0.9102	0.024*	
C2	0.5383 (2)	0.36093 (14)	0.97290 (9)	0.0258 (4)	
H2A	0.5983	0.3382	1.0130	0.031*	
H2B	0.4949	0.4229	0.9859	0.031*	
C3	0.4122 (3)	0.29143 (15)	0.95935 (10)	0.0314 (5)	
H3A	0.3420	0.2917	0.9994	0.038*	
H3B	0.4534	0.2268	0.9546	0.038*	
C4	0.3289 (2)	0.31858 (13)	0.89248 (10)	0.0253 (4)	
H4	0.2426	0.2743	0.8867	0.030*	
C5	0.4337 (2)	0.30608 (12)	0.82959 (9)	0.0196 (4)	
H5	0.4687	0.2391	0.8254	0.024*	
C6	0.3504 (2)	0.33800 (12)	0.76349 (9)	0.0183 (4)	
H6	0.2420	0.3455	0.7752	0.022*	
C7	0.4165 (2)	0.43769 (12)	0.74658 (9)	0.0162 (3)	
C8	0.53840 (19)	0.43831 (12)	0.69037 (9)	0.0158 (3)	
C9	0.6448 (2)	0.35546 (11)	0.70054 (8)	0.0158 (3)	
H9	0.5985	0.2951	0.6840	0.019*	
C10	0.6892 (2)	0.34851 (12)	0.78001 (9)	0.0168 (3)	
H10	0.7135	0.2804	0.7886	0.020*	
C11	0.5674 (2)	0.37552 (11)	0.83597 (9)	0.0164 (3)	
C12	0.84195 (19)	0.40274 (13)	0.78403 (9)	0.0195 (4)	
H12A	0.8383	0.4521	0.8207	0.023*	
H12B	0.9244	0.3584	0.7950	0.023*	
C13	0.8657 (2)	0.44761 (12)	0.71208 (9)	0.0172 (3)	
H13	0.9745	0.4553	0.7017	0.021*	
C14	0.7952 (2)	0.37223 (12)	0.66496 (9)	0.0169 (3)	
H14	0.8562	0.3130	0.6676	0.020*	
C15	0.6204 (2)	0.53451 (12)	0.68240 (9)	0.0176 (3)	
H15A	0.6172	0.5522	0.6323	0.021*	
H15B	0.5617	0.5826	0.7082	0.021*	
C16	0.7835 (2)	0.54252 (12)	0.70678 (9)	0.0174 (3)	
H16	0.7850	0.5735	0.7538	0.021*	
C17	0.4825 (2)	0.46807 (12)	0.81829 (9)	0.0160 (3)	
H17	0.5558	0.5206	0.8112	0.019*	
C19	0.2676 (2)	0.42064 (14)	0.89324 (11)	0.0254 (4)	
H19A	0.1831	0.4248	0.8600	0.030*	
H19B	0.2282	0.4346	0.9405	0.030*	
C21	0.3093 (2)	0.58623 (13)	0.86550 (10)	0.0239 (4)	
H21A	0.2231	0.5803	0.8333	0.029*	
H21B	0.3826	0.6297	0.8437	0.029*	
C22	0.2559 (3)	0.62839 (16)	0.93459 (11)	0.0325 (5)	
H22A	0.3375	0.6265	0.9688	0.049*	
H22B	0.1710	0.5917	0.9522	0.049*	
H22C	0.2250	0.6941	0.9272	0.049*	
C23	0.8432 (2)	0.45474 (15)	0.96600 (10)	0.0280 (4)	
H23A	0.9164	0.4092	0.9480	0.042*	
H23B	0.8064	0.4333	1.0116	0.042*	
H23C	0.8907	0.5168	0.9711	0.042*	
C24	0.3230 (2)	0.49072 (13)	0.64212 (9)	0.0222 (4)	
H24A	0.2297	0.4661	0.6211	0.027*	
H24B	0.3448	0.5534	0.6213	0.027*	
C25	0.9100 (2)	0.40192 (14)	0.55639 (9)	0.0235 (4)	
H25A	0.9603	0.3404	0.5580	0.035*	
H25B	0.9741	0.4500	0.5779	0.035*	
H25C	0.8902	0.4191	0.5076	0.035*	
C26	0.9849 (2)	0.64858 (14)	0.68263 (12)	0.0309 (5)	
H26A	1.0587	0.5996	0.6938	0.046*	
H26B	0.9618	0.6849	0.7249	0.046*	
H26C	1.0256	0.6910	0.6468	0.046*	
C27	0.2532 (2)	0.26281 (12)	0.66158 (10)	0.0217 (4)	
C28	0.2948 (3)	0.20041 (14)	0.60156 (10)	0.0290 (4)	
H28A	0.2156	0.2024	0.5662	0.043*	
H28B	0.3074	0.1353	0.6182	0.043*	
H28C	0.3888	0.2226	0.5809	0.043*	

### Atomic displacement parameters (Å^2^)

**Table:**

	*U*^11^	*U*^22^	*U*^33^	*U*^12^	*U*^13^	*U*^23^
O1	0.0192 (6)	0.0210 (6)	0.0201 (6)	0.0050 (5)	−0.0024 (5)	0.0006 (5)
O2	0.0209 (6)	0.0231 (6)	0.0169 (6)	0.0025 (5)	−0.0042 (5)	−0.0019 (5)
O3	0.0232 (6)	0.0172 (6)	0.0192 (6)	−0.0015 (5)	−0.0048 (5)	−0.0015 (5)
O4	0.0214 (6)	0.0166 (6)	0.0205 (6)	−0.0012 (5)	−0.0026 (5)	−0.0049 (5)
O5	0.0222 (7)	0.0412 (8)	0.0355 (8)	0.0049 (7)	−0.0078 (6)	−0.0115 (7)
O7	0.0208 (6)	0.0235 (6)	0.0139 (6)	0.0023 (5)	−0.0001 (5)	−0.0004 (5)
O8	0.0231 (6)	0.0188 (6)	0.0277 (7)	−0.0045 (6)	0.0002 (6)	0.0053 (5)
N1	0.0202 (7)	0.0173 (7)	0.0196 (7)	−0.0008 (6)	0.0042 (6)	−0.0032 (6)
C1	0.0277 (9)	0.0134 (7)	0.0180 (8)	0.0003 (7)	−0.0022 (8)	0.0013 (6)
C2	0.0372 (11)	0.0245 (9)	0.0157 (9)	−0.0057 (9)	0.0001 (8)	0.0021 (7)
C3	0.0460 (12)	0.0281 (10)	0.0200 (9)	−0.0140 (10)	0.0045 (9)	0.0034 (8)
C4	0.0310 (10)	0.0225 (9)	0.0223 (9)	−0.0124 (8)	0.0046 (8)	−0.0007 (7)
C5	0.0260 (9)	0.0136 (8)	0.0191 (9)	−0.0036 (7)	−0.0001 (7)	−0.0005 (6)
C6	0.0199 (8)	0.0156 (8)	0.0194 (8)	−0.0026 (7)	0.0002 (7)	−0.0032 (7)
C7	0.0157 (8)	0.0141 (7)	0.0187 (8)	0.0005 (7)	−0.0007 (7)	0.0010 (6)
C8	0.0187 (8)	0.0147 (8)	0.0140 (8)	0.0011 (7)	−0.0021 (7)	−0.0004 (6)
C9	0.0184 (8)	0.0136 (7)	0.0154 (8)	0.0008 (7)	−0.0009 (7)	−0.0007 (6)
C10	0.0203 (8)	0.0136 (7)	0.0166 (8)	0.0037 (7)	−0.0008 (7)	0.0006 (6)
C11	0.0212 (8)	0.0120 (7)	0.0159 (8)	−0.0002 (7)	−0.0002 (7)	0.0011 (6)
C12	0.0184 (8)	0.0216 (8)	0.0185 (8)	0.0018 (7)	−0.0024 (7)	0.0020 (7)
C13	0.0183 (7)	0.0169 (8)	0.0166 (8)	0.0027 (7)	−0.0007 (7)	−0.0009 (6)
C14	0.0195 (8)	0.0151 (8)	0.0160 (8)	0.0026 (7)	−0.0003 (7)	−0.0011 (6)
C15	0.0196 (9)	0.0151 (8)	0.0181 (8)	0.0030 (7)	0.0006 (7)	0.0025 (6)
C16	0.0203 (8)	0.0150 (8)	0.0171 (8)	−0.0003 (7)	0.0012 (7)	0.0006 (6)
C17	0.0181 (8)	0.0141 (7)	0.0158 (8)	−0.0008 (7)	0.0019 (7)	−0.0009 (6)
C19	0.0255 (9)	0.0284 (10)	0.0223 (10)	−0.0073 (8)	0.0071 (8)	−0.0042 (7)
C21	0.0242 (9)	0.0237 (9)	0.0240 (9)	0.0048 (8)	0.0002 (8)	−0.0037 (7)
C22	0.0350 (11)	0.0320 (11)	0.0304 (11)	0.0120 (9)	0.0020 (9)	−0.0085 (8)
C23	0.0269 (10)	0.0335 (10)	0.0237 (9)	0.0028 (9)	−0.0071 (8)	−0.0031 (8)
C24	0.0216 (9)	0.0247 (9)	0.0204 (9)	0.0032 (8)	−0.0043 (7)	0.0005 (7)
C25	0.0234 (8)	0.0292 (10)	0.0180 (9)	−0.0001 (8)	0.0025 (7)	0.0015 (7)
C26	0.0256 (10)	0.0214 (9)	0.0457 (12)	−0.0053 (8)	−0.0010 (9)	−0.0005 (9)
C27	0.0228 (9)	0.0188 (8)	0.0236 (9)	−0.0038 (8)	−0.0021 (7)	−0.0003 (7)
C28	0.0353 (11)	0.0275 (10)	0.0242 (10)	0.0037 (9)	−0.0070 (9)	−0.0063 (8)

### Geometric parameters (Å, °)

**Table:**

O1—C7	1.433 (2)	C10—C12	1.568 (2)
O1—C24	1.438 (2)	C10—C11	1.574 (2)
O2—C24	1.409 (2)	C10—H10	1.0000
O2—C8	1.461 (2)	C11—C17	1.550 (2)
O3—C23	1.424 (2)	C12—C13	1.529 (2)
O3—C1	1.435 (2)	C12—H12A	0.9900
O4—C27	1.354 (2)	C12—H12B	0.9900
O4—C6	1.449 (2)	C13—C14	1.531 (2)
O5—C27	1.198 (2)	C13—C16	1.533 (2)
O7—C25	1.419 (2)	C13—H13	1.0000
O7—C14	1.422 (2)	C14—H14	1.0000
O8—C26	1.422 (2)	C15—C16	1.536 (2)
O8—C16	1.438 (2)	C15—H15A	0.9900
N1—C21	1.466 (2)	C15—H15B	0.9900
N1—C17	1.468 (2)	C16—H16	1.0000
N1—C19	1.469 (2)	C17—H17	1.0000
C1—C2	1.534 (3)	C19—H19A	0.9900
C1—C11	1.557 (2)	C19—H19B	0.9900
C1—H1	1.0000	C21—C22	1.525 (3)
C2—C3	1.518 (3)	C21—H21A	0.9900
C2—H2A	0.9900	C21—H21B	0.9900
C2—H2B	0.9900	C22—H22A	0.9800
C3—C4	1.528 (3)	C22—H22B	0.9800
C3—H3A	0.9900	C22—H22C	0.9800
C3—H3B	0.9900	C23—H23A	0.9800
C4—C5	1.534 (3)	C23—H23B	0.9800
C4—C19	1.543 (3)	C23—H23C	0.9800
C4—H4	1.0000	C24—H24A	0.9900
C5—C6	1.534 (2)	C24—H24B	0.9900
C5—C11	1.552 (2)	C25—H25A	0.9800
C5—H5	1.0000	C25—H25B	0.9800
C6—C7	1.562 (2)	C25—H25C	0.9800
C6—H6	1.0000	C26—H26A	0.9800
C7—C8	1.531 (2)	C26—H26B	0.9800
C7—C17	1.553 (2)	C26—H26C	0.9800
C8—C9	1.522 (2)	C27—C28	1.494 (3)
C8—C15	1.552 (2)	C28—H28A	0.9800
C9—C14	1.526 (2)	C28—H28B	0.9800
C9—C10	1.573 (2)	C28—H28C	0.9800
C9—H9	1.0000		
			
C7—O1—C24	105.84 (13)	C12—C13—C14	100.56 (14)
C24—O2—C8	103.92 (13)	C12—C13—C16	110.85 (14)
C23—O3—C1	113.26 (14)	C14—C13—C16	111.90 (14)
C27—O4—C6	116.41 (14)	C12—C13—H13	111.0
C25—O7—C14	111.81 (14)	C14—C13—H13	111.0
C26—O8—C16	113.22 (15)	C16—C13—H13	111.0
C21—N1—C17	113.23 (14)	O7—C14—C9	109.93 (14)
C21—N1—C19	111.68 (15)	O7—C14—C13	117.06 (14)
C17—N1—C19	115.87 (14)	C9—C14—C13	102.05 (13)
O3—C1—C2	108.29 (14)	O7—C14—H14	109.1
O3—C1—C11	108.02 (13)	C9—C14—H14	109.1
C2—C1—C11	117.18 (16)	C13—C14—H14	109.1
O3—C1—H1	107.7	C16—C15—C8	118.92 (14)
C2—C1—H1	107.7	C16—C15—H15A	107.6
C11—C1—H1	107.7	C8—C15—H15A	107.6
C3—C2—C1	113.19 (16)	C16—C15—H15B	107.6
C3—C2—H2A	108.9	C8—C15—H15B	107.6
C1—C2—H2A	108.9	H15A—C15—H15B	107.0
C3—C2—H2B	108.9	O8—C16—C13	112.28 (14)
C1—C2—H2B	108.9	O8—C16—C15	104.50 (14)
H2A—C2—H2B	107.8	C13—C16—C15	114.26 (14)
C2—C3—C4	110.01 (16)	O8—C16—H16	108.5
C2—C3—H3A	109.7	C13—C16—H16	108.5
C4—C3—H3A	109.7	C15—C16—H16	108.5
C2—C3—H3B	109.7	N1—C17—C11	110.66 (14)
C4—C3—H3B	109.7	N1—C17—C7	118.36 (14)
H3A—C3—H3B	108.2	C11—C17—C7	98.35 (13)
C3—C4—C5	109.20 (17)	N1—C17—H17	109.6
C3—C4—C19	113.58 (17)	C11—C17—H17	109.6
C5—C4—C19	109.38 (14)	C7—C17—H17	109.6
C3—C4—H4	108.2	N1—C19—C4	114.17 (16)
C5—C4—H4	108.2	N1—C19—H19A	108.7
C19—C4—H4	108.2	C4—C19—H19A	108.7
C4—C5—C6	108.35 (15)	N1—C19—H19B	108.7
C4—C5—C11	109.64 (14)	C4—C19—H19B	108.7
C6—C5—C11	104.64 (13)	H19A—C19—H19B	107.6
C4—C5—H5	111.3	N1—C21—C22	112.21 (16)
C6—C5—H5	111.3	N1—C21—H21A	109.2
C11—C5—H5	111.3	C22—C21—H21A	109.2
O4—C6—C5	109.86 (14)	N1—C21—H21B	109.2
O4—C6—C7	116.54 (14)	C22—C21—H21B	109.2
C5—C6—C7	104.58 (14)	H21A—C21—H21B	107.9
O4—C6—H6	108.5	C21—C22—H22A	109.5
C5—C6—H6	108.5	C21—C22—H22B	109.5
C7—C6—H6	108.5	H22A—C22—H22B	109.5
O1—C7—C8	101.93 (13)	C21—C22—H22C	109.5
O1—C7—C17	116.49 (14)	H22A—C22—H22C	109.5
C8—C7—C17	110.30 (14)	H22B—C22—H22C	109.5
O1—C7—C6	111.66 (14)	O3—C23—H23A	109.5
C8—C7—C6	114.84 (14)	O3—C23—H23B	109.5
C17—C7—C6	102.17 (13)	H23A—C23—H23B	109.5
O2—C8—C9	113.19 (14)	O3—C23—H23C	109.5
O2—C8—C7	98.17 (13)	H23A—C23—H23C	109.5
C9—C8—C7	110.58 (14)	H23B—C23—H23C	109.5
O2—C8—C15	106.73 (13)	O2—C24—O1	107.87 (14)
C9—C8—C15	113.04 (14)	O2—C24—H24A	110.1
C7—C8—C15	114.22 (14)	O1—C24—H24A	110.1
C8—C9—C14	112.02 (14)	O2—C24—H24B	110.1
C8—C9—C10	109.23 (13)	O1—C24—H24B	110.1
C14—C9—C10	102.55 (14)	H24A—C24—H24B	108.4
C8—C9—H9	110.9	O7—C25—H25A	109.5
C14—C9—H9	110.9	O7—C25—H25B	109.5
C10—C9—H9	110.9	H25A—C25—H25B	109.5
C12—C10—C9	103.69 (13)	O7—C25—H25C	109.5
C12—C10—C11	116.83 (14)	H25A—C25—H25C	109.5
C9—C10—C11	117.78 (14)	H25B—C25—H25C	109.5
C12—C10—H10	105.8	O8—C26—H26A	109.5
C9—C10—H10	105.8	O8—C26—H26B	109.5
C11—C10—H10	105.8	H26A—C26—H26B	109.5
C17—C11—C5	98.02 (14)	O8—C26—H26C	109.5
C17—C11—C1	114.49 (14)	H26A—C26—H26C	109.5
C5—C11—C1	113.19 (14)	H26B—C26—H26C	109.5
C17—C11—C10	113.31 (13)	O5—C27—O4	123.60 (17)
C5—C11—C10	109.07 (13)	O5—C27—C28	125.38 (18)
C1—C11—C10	108.45 (14)	O4—C27—C28	111.02 (16)
C13—C12—C10	106.23 (14)	C27—C28—H28A	109.5
C13—C12—H12A	110.5	C27—C28—H28B	109.5
C10—C12—H12A	110.5	H28A—C28—H28B	109.5
C13—C12—H12B	110.5	C27—C28—H28C	109.5
C10—C12—H12B	110.5	H28A—C28—H28C	109.5
H12A—C12—H12B	108.7	H28B—C28—H28C	109.5
			
C23—O3—C1—C2	78.45 (18)	C2—C1—C11—C10	−157.24 (15)
C23—O3—C1—C11	−153.71 (15)	C12—C10—C11—C17	79.16 (18)
O3—C1—C2—C3	160.33 (16)	C9—C10—C11—C17	−45.3 (2)
C11—C1—C2—C3	37.9 (2)	C12—C10—C11—C5	−172.79 (14)
C1—C2—C3—C4	−52.0 (2)	C9—C10—C11—C5	62.72 (18)
C2—C3—C4—C5	66.2 (2)	C12—C10—C11—C1	−49.12 (18)
C2—C3—C4—C19	−56.2 (2)	C9—C10—C11—C1	−173.60 (14)
C3—C4—C5—C6	−177.44 (15)	C9—C10—C12—C13	7.26 (17)
C19—C4—C5—C6	−52.58 (19)	C11—C10—C12—C13	−124.09 (15)
C3—C4—C5—C11	−63.79 (18)	C10—C12—C13—C14	−34.41 (16)
C19—C4—C5—C11	61.07 (19)	C10—C12—C13—C16	84.09 (17)
C27—O4—C6—C5	153.01 (15)	C25—O7—C14—C9	−173.13 (14)
C27—O4—C6—C7	−88.33 (19)	C25—O7—C14—C13	71.10 (19)
C4—C5—C6—O4	−130.98 (14)	C8—C9—C14—O7	−53.00 (18)
C11—C5—C6—O4	112.10 (15)	C10—C9—C14—O7	−170.00 (13)
C4—C5—C6—C7	103.22 (16)	C8—C9—C14—C13	71.91 (16)
C11—C5—C6—C7	−13.69 (17)	C10—C9—C14—C13	−45.09 (15)
C24—O1—C7—C8	28.66 (17)	C12—C13—C14—O7	169.43 (15)
C24—O1—C7—C17	148.75 (15)	C16—C13—C14—O7	51.7 (2)
C24—O1—C7—C6	−94.42 (16)	C12—C13—C14—C9	49.40 (15)
O4—C6—C7—O1	92.28 (17)	C16—C13—C14—C9	−68.33 (17)
C5—C6—C7—O1	−146.23 (14)	O2—C8—C15—C16	−143.22 (15)
O4—C6—C7—C8	−23.1 (2)	C9—C8—C15—C16	−18.1 (2)
C5—C6—C7—C8	98.38 (16)	C7—C8—C15—C16	109.45 (17)
O4—C6—C7—C17	−142.51 (15)	C26—O8—C16—C13	−76.69 (18)
C5—C6—C7—C17	−21.02 (16)	C26—O8—C16—C15	158.94 (14)
C24—O2—C8—C9	160.42 (14)	C12—C13—C16—O8	153.62 (14)
C24—O2—C8—C7	43.82 (15)	C14—C13—C16—O8	−94.99 (17)
C24—O2—C8—C15	−74.60 (16)	C12—C13—C16—C15	−87.59 (18)
O1—C7—C8—O2	−44.26 (15)	C14—C13—C16—C15	23.8 (2)
C17—C7—C8—O2	−168.60 (13)	C8—C15—C16—O8	144.03 (15)
C6—C7—C8—O2	76.63 (16)	C8—C15—C16—C13	20.9 (2)
O1—C7—C8—C9	−162.87 (13)	C21—N1—C17—C11	172.51 (14)
C17—C7—C8—C9	72.79 (17)	C19—N1—C17—C11	−56.6 (2)
C6—C7—C8—C9	−41.98 (19)	C21—N1—C17—C7	−75.16 (19)
O1—C7—C8—C15	68.29 (17)	C19—N1—C17—C7	55.8 (2)
C17—C7—C8—C15	−56.06 (18)	C5—C11—C17—N1	69.33 (16)
C6—C7—C8—C15	−170.82 (14)	C1—C11—C17—N1	−50.74 (19)
O2—C8—C9—C14	92.06 (17)	C10—C11—C17—N1	−175.83 (14)
C7—C8—C9—C14	−158.92 (14)	C5—C11—C17—C7	−55.31 (14)
C15—C8—C9—C14	−29.44 (19)	C1—C11—C17—C7	−175.38 (14)
O2—C8—C9—C10	−155.03 (14)	C10—C11—C17—C7	59.53 (16)
C7—C8—C9—C10	−46.01 (18)	O1—C7—C17—N1	50.6 (2)
C15—C8—C9—C10	83.48 (17)	C8—C7—C17—N1	166.10 (14)
C8—C9—C10—C12	−96.06 (16)	C6—C7—C17—N1	−71.36 (17)
C14—C9—C10—C12	22.92 (16)	O1—C7—C17—C11	169.57 (14)
C8—C9—C10—C11	34.7 (2)	C8—C7—C17—C11	−74.93 (15)
C14—C9—C10—C11	153.71 (14)	C6—C7—C17—C11	47.61 (15)
C4—C5—C11—C17	−73.03 (16)	C21—N1—C19—C4	170.95 (16)
C6—C5—C11—C17	43.00 (16)	C17—N1—C19—C4	39.3 (2)
C4—C5—C11—C1	48.02 (19)	C3—C4—C19—N1	81.9 (2)
C6—C5—C11—C1	164.04 (14)	C5—C4—C19—N1	−40.3 (2)
C4—C5—C11—C10	168.83 (14)	C17—N1—C21—C22	−156.45 (16)
C6—C5—C11—C10	−75.14 (16)	C19—N1—C21—C22	70.6 (2)
O3—C1—C11—C17	−47.39 (19)	C8—O2—C24—O1	−28.12 (17)
C2—C1—C11—C17	75.15 (19)	C7—O1—C24—O2	−1.39 (18)
O3—C1—C11—C5	−158.61 (14)	C6—O4—C27—O5	−11.0 (3)
C2—C1—C11—C5	−36.1 (2)	C6—O4—C27—C28	168.90 (15)
O3—C1—C11—C10	80.22 (16)		
